# A gene transcription signature associated with hormone independence in a subset of both breast and prostate cancers

**DOI:** 10.1186/1471-2164-8-199

**Published:** 2007-06-28

**Authors:** Chad J Creighton

**Affiliations:** 1 Department of Medicine, Dan L. Duncan Cancer Center Division of Biostatistics, Baylor College of Medicine, Houston, TX 77030, USA

## Abstract

**Background:**

The development of resistance to hormone therapy in both breast and prostate cancers is attributed to tens of thousands of patient deaths every year.

**Results:**

From analyses of global gene expression profile data, a nonrandom amount of overlap was observed between the set of genes associated with estrogen receptor negative (ER-), hormone independent breast cancer and the set of genes associated with androgen independent (AI) prostate cancer. A set of 81 genes was identified that were differentially expressed between ER- and ER+ clinical breast tumors and breast cancer cell lines and that showed concordant expression in AI versus AS (androgen sensitive) prostate cell lines. This common gene signature of hormone independence was used to identify a subset of clinically localized primary prostate tumors that shared extensive similarities in gene transcription with both ER- breast and AI prostate cell lines and that tended to show concurrent deactivation of the androgen signaling pathway. Both ER- breast and AI prostate cell lines were significantly enriched for transcriptional targets of signaling via epidermal growth factor receptor (EGFR).

**Conclusion:**

This study indicates that the growth- and survival-promoting functions of hormone receptors can be bypassed in a subset of both breast and prostate cancers by the same growth factor signaling pathways, which holds implications for the use of targeted therapy regimens.

## Background

In 2006, on the order of 234,000 men and 213,000 women were diagnosed with prostate cancer and breast cancer, respectively, and about 27,000 men and 41,000 women died (American Cancer Society statistics). Steroid hormone receptor signaling has been linked to all stages of prostate and breast carcinogenesis [1,2]. Initial treatment of clinically localized prostate cancer (PCA) and invasive breast cancer (IBC) usually involves surgical removal of the cancerous tissue or radiation therapy. The clinical use of adjuvant anti-androgen therapy in PCA and of anti-estrogen therapy in IBC has aided greatly in prolonging or preventing disease recurrence, as the majority of these cancers, at least initially, depend upon their associated hormones for growth. However, significant fractions of PCA and IBC either initially present as hormone independent or develop hormone independence over the course of anti-hormone therapy [3,4]. In the case of IBC, hormone independence correlates closely with expression of the estrogen receptor alpha (ER), with 30–35% of IBC being ER-negative (ER-) and exhibiting no requirement of estrogen for growth [3,5]. Except for the fraction of ER- IBC that express HER2/neu, no targeted therapy is currently in widespread use for ER- IBC. In the case of advanced PCA, androgen ablation therapy effectively results in tumor regression over the short-term; in most cases, however, the recurrence of highly aggressive and metastatic prostate cancer that is resistant to hormone therapy occurs as a result [1,4].

Breast and prostate cancers share much in common with each other, in that they both manifest as either hormone dependent or independent. The hypothesis explored in this present study is that the molecular mechanisms of acquirement of hormone independence are similar between IBC and PCA [4]. Global gene expression profiling studies, carried out in breast and prostate cancers separately, indicate that on the order of hundreds or even thousands of genes might be involved in hormone independence in each disease [6-8]. If a select set of genes common to hormone independent breast and prostate cancers could be identified, it might be indicative of a core transcriptional program on which attention could be focused. The main strategy of this study was to look for patterns of *enrichment*, i.e. to look for a non-random amount of shared overlap between distinct sets of genes associated separately with either breast or prostate cancers. Such a pattern of enrichment may involve only a fraction of the genes from each cancer type and yet may hold biological and clinical significance.

## Results

### A gene expression signature of ER-, hormone-independent clinical breast tumors that is partially manifested in ER- breast cancer cell lines

The basic approach of this study was to first derive separate gene expression signature patterns of hormone independence from breast and prostate cancers and then to determine whether the two signatures shared significant similarity with each other. A gene transcription signature of ER- (hormone-independent) versus ER+ (hormone-dependent) invasive breast cancer (IBC) was defined by selecting genes showing differential expression (*p*<0.01) in each of two independent mRNA profile datasets of 295 clinical IBC (the dataset from van de Vijver *et al*., ref [9], with 69 profiles from ER- tumors) and 286 tumors (the dataset from Wang *et al*., ref [10], with 77 ER- profiles). Of the 2486 uniquely identified genes in this ER-status signature, 1332 were higher in the ER- tumors.

**Figure 1:**
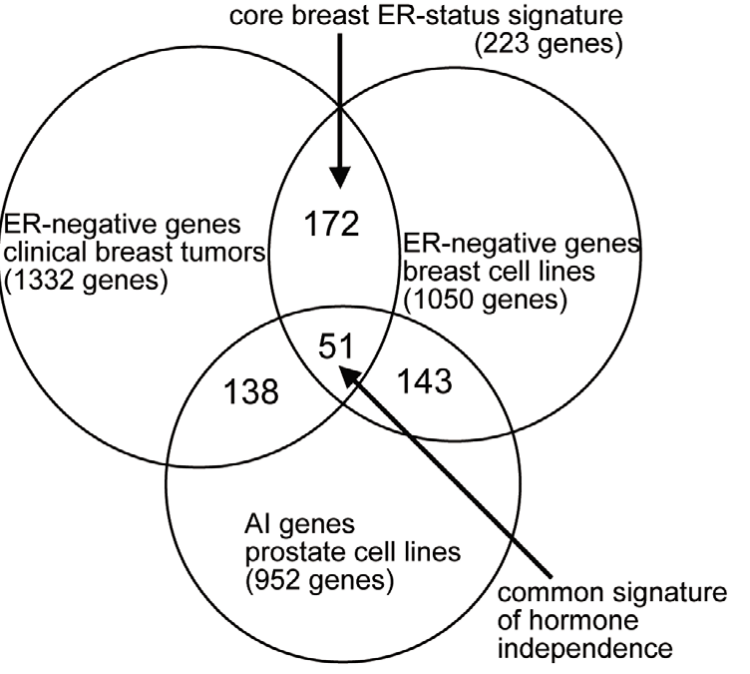
Genes associated with hormone independence in breast cancer share significant overlap with genes associated with hormone independence in prostate cancer. Venn diagram showing the overlap between the following sets of genes: (1) genes more highly expressed in clinical ER- over ER+ breast tumors (*p*<0.01 in each of the RNA profile datasets from van de Vijver *et al*. and Wang *et al*.), (2) genes more highly expressed in ER- over ER+ cell lines (*p*<0.01 in the profile dataset from Bild *et al*.), and (3) genes more highly expressed in androgen independent (AI) over androgen sensitive (AS) prostate cell lines (*p*<0.05 in the dataset from Zhao *et al*.). *Core breast ER-status signature*, genes shared between the clinical breast tumor and breast cancer cell line sets. *Common signature of hormone independence*, genes shared between all three sets.

The expression patterns of the ER-status gene signature as derived from clinical IBC were further examined in an additional dataset of breast cancer cell lines from Bild *et al*. [11], which consisted of 28 mRNA profiles representing 18 different cell lines (ten of them ER-). As expected, a significant portion of the clinical ER-status signature showed the corresponding expression patterns in cell lines. Out of the 1332 genes found to be higher in ER- clinical tumors, 223 were higher in ER- cell lines (*p*<0.01), while 848 showed no such trend (*p*>0.1). The intersection of the clinical and cell line breast cancer signatures was termed the "core breast ER-status signature" and consisted of 223 ER- genes (Venn diagram represented in Figure 1) and 194 ER+ genes (*p*<0.01 in each of Bild, van de Vijver, and Wang datasets). This core signature pattern was considered to be independent of the tissue or environmental context. The suggestion that a set of genes associated with hormone independence in breast cancer were regulated *in vivo *but not *in vitro *seemed intriguing but was not further explored in this study.

**Figure 2:**
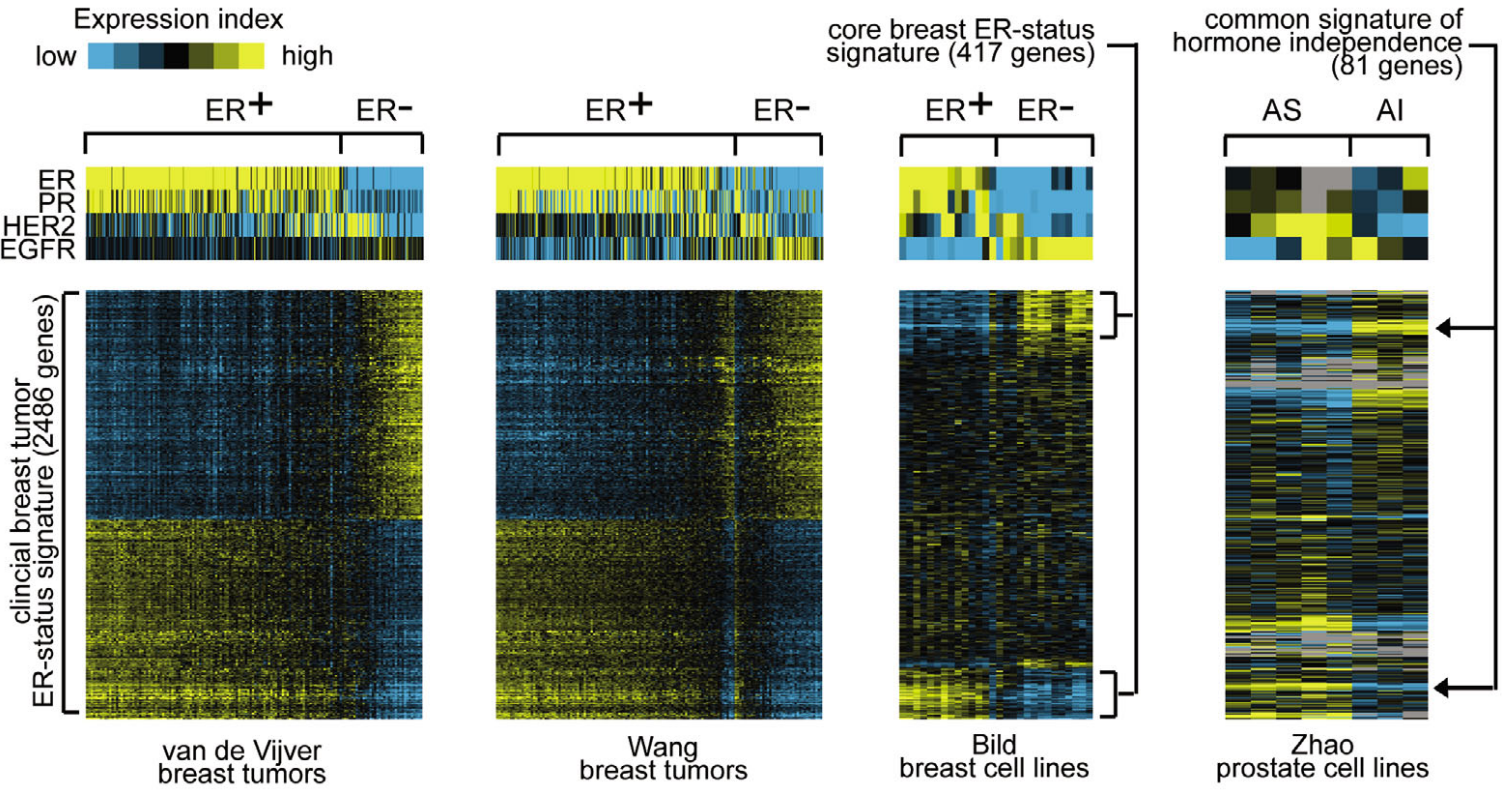
Gene expression patterns of ER- clinical breast cancer are observed in both breast and prostate cancer cell lines. Heat map representation for 2486 unique named genes differentially expressed between ER+ and ER- breast tumors (*p*<0.01 in both van de Vijver and Wang RNA profile datasets, 1332 higher in ER-). Expression patterns are represented as a color map. Each row represents a gene; each column represents a sample. The level of expression of each gene in each sample is represented using a yellow-blue color scale (yellow: high expression). Patterns corresponding to the 2486 genes are shown in both the Bild profile dataset of 18 breast cancer cell lines (ten ER-) and the Zhao dataset of eight prostate cell lines (AS, androgen sensitive; AI, androgen independent; gray denotes missing values or unrepresented genes). The order of the genes is the same for each of the datasets. Corresponding expression patterns for genes *ER*, *PR*, *HER2*, and *EGFR *are also shown. The order of the breast and prostate cell lines profiles is the same as that for Figure 4 (where they are labeled by name). Genes and associated expression values are available in Additional File 1.

The expression patterns of the clinical breast ER-status signature were visualized as heat maps in both the clinical and cell culture profile datasets (Figure 2). From the heat map representation, it was apparent that a small fraction of the breast tumors in the Wang profile dataset that were classified as ER+ by immunohistochemistry showed gene expression patterns more characteristic of ER- tumors, as well as low ER mRNA. Similarly, one particular breast cancer cell line, HCC1428, was designated as ER+ [12] but from its profile appeared more similar to ER- cell lines. In the heat map representation (Figure 2), the ER- tumor and cell line profiles were ordered by increasing similarity to the overall ER- expression pattern. The small fraction of ER- tumors which did not fit the pattern tended to have high RNA expression of the *HER2 *oncogene. In addition, the ER-, HER2+ SKBR3 cell line did not fit the pattern of the other ER- cell lines. All of this indicated that the core breast ER-status signature (Figure 2) was a pattern of ER-, HER2- breast cancer, with the ER-, HER2+ breast cancers having a different pattern, as has been indicated in previous expression profiling studies [6,7,12].

### A gene expression signature common to ER- breast cancer and AI, hormone-independent prostate cancer cell lines

Unlike breast cancer, there are currently no well-defined molecular markers of hormone-independent prostate cancer. For example, expression of the androgen receptor (AR) does not appear to correlate with response to hormone therapy in prostate cancer, and AR protein is expressed fairly homogeneously in primary tumors, recurrent local tumors, and metastases [1]. It was therefore difficult to define a signature of hormone-independence from profiles of clinical prostate tumors alone. However, a number of prostate cancer cell lines have been established that are classified as either androgen sensitive (AS), which respond to androgen stimulation, and androgen insensitive (AI), which do not respond. As discussed below (see Discussion and [4]), there are several known mechanisms by which prostate cancers may develop resistance to hormone therapy. While the cell lines considered here were entirely androgen pathway independent, other prostate cancers develop hypersensitivity to androgen receptor pathway signaling; this latter type of hormone therapy resistant cancer was not considered in this study.

From an mRNA profile data of eight different prostate cell lines, three of them AI, a set of 1793 genes that showed differential expression (*p*<0.05) between AS and AI was obtained, 952 of these genes being higher in AI. The overlap of these 952 AI genes with the 223 core breast ER- signature (Figure 1) was 51; by chance, around 19 genes would have been expected to overlap, which makes the observed overlap of 51 highly significant (*p *= 1E-11, one-sided Fisher's exact). A list of the 51 common prostate AI/breast ER- genes is provided in Table 1 (heat map representation in Figure 2, associated gene expression values provided in Additional File 1). Only three genes in the list (*KNTC2*, *EXT1*, and *CDC25B*) were annotated by Gene Ontology as having roles in the cell cycle or cell division, and so the 51 genes as a group do not appear to represent a program of general cellular proliferation.

**Table 1:** Genes with elevated mRNA levels in common signature of hormone independence (Figure 1)

Entrez	Name	Title	Entrez	Name	Title
87	ACTN1	actinin, alpha 1	5329	PLAUR	plasminogen activator, urokinase receptor
136	ADORA2B	adenosine A2b receptor	5359	PLSCR1	phospholipid scramblase 1
390	ARHE	Rho family GTPase 3	5621	PRNP	Prion protein (p27–30)
824	CAPN2	calpain 2, (m/II) large subunit	6732	SRPK1	SFRS protein kinase 1
858	CAV2	caveolin 2	7272	TTK	TTK protein kinase
994	CDC25B	cell division cycle 25B	7296	TXNRD1	thioredoxin reductase 1
1075	CTSC	cathepsin C	7378	UP	uridine phosphorylase 1
1284	COL4A2	collagen, type IV, alpha 2	7398	USP1	ubiquitin specific peptidase 1
1786	DNMT1	DNA (cytosine-5-)-methyltransferase 1	8882	ZNF259	zinc finger protein 259
1969	EPHA2	EPH receptor A2	8898	MTMR2	myotubularin related protein 2
2000	ELF4	E74-like factor 4 (ets domain)	9056	SLC7A7	solute carrier family 7, member 7
2023	ENO1	enolase 1, (alpha)	9322	TRIP10	thyroid hormone receptor interactor 10
2037	EPB41L2	erythrocyte membrane protein band 4.1-like 2	10403	KNTC2	kinetochore associated 2
2131	EXT1	exostoses (multiple) 1	10479	SLC9A6	solute carrier family 9, member 6
2182	ACSL4	acyl-CoA synthetase long-chain member 4	10644	IMP-2	IGF-II mRNA-binding protein 2
2633	GBP1	guanylate binding protein 1, interferon-inducible	10946	SF3A3	splicing factor 3a, subunit 3, 60 kDa
2920	CXCL1	chemokine (C-X-C motif) ligand 2	25937	DKFZP586I1419	WW domain containing transcription regulator 1
3383	ICAM1	intercellular adhesion molecule 1 (CD54), human rhinovirus receptor	26031	OSBPL3	oxysterol binding protein-like 3
3569	IL6	interleukin 6 (interferon, beta 2)	26064	RAI14	retinoic acid induced 14
3575	IL7R	interleukin 7 receptor	29083	HSPC135	HSPC135 protein
3600	IL15	interleukin 15	29970	SCHIP1	schwannomin interacting protein 1
3801	KIFC3	kinesin family member C3	29980	DONSON	downstream neighbor of SON
3934	LCN2	lipocalin 2 (oncogene 24p3)	55003	PAK1IP1	PAK1 interacting protein 1
4478	MSN	moesin	56913	C1GALT1	glycoprotein-N-acetylgalactosamine 3-beta-galactosyltransferase
4907	NT5E	5'-nucleotidase, ecto (CD73)	140885	PTPNS1	protein tyrosine phosphatase, non-receptor type substrate 1
5271	SERPINB8	serpin peptidase inhibitor, clade B			

The 952 prostate AI genes also shared highly significant overlap with the breast ER- clinical and breast ER- cell line gene sets individually (189 genes, Fisher's exact *p *= 1E-16; and 194 genes, *p *= 1E-39, respectively, see Figure 1). Similarly, the 841 genes higher in AS over AI prostate cell lines shared a highly significant overlap of 30 with the set of 194 in the core breast ER+ signature (expected 11, Fisher's exact *p *= 1E-07). A list of these 30 common prostate AS/breast ER+ genes is provided in Table 2. Of the 30 genes, six (*CISH*, *KIAA0182*, *ICA1*, *PGR*, *SLC1A4*, *XBP1*) were up-regulated by estrogen signaling (in cluster "B" from ref **13**) and six (*KIAA0182*, *GLUD1*, *FOXA1*, *FXYD3*, *NPDC1*, *HIST2H2BE*) were up-regulated (*p*<0.001) by androgen signaling, based on analysis of data from published RNA profiling studies of breast cancer [13] and prostate cancer [14] cell cultures, respectively.

**Table 2:** Genes with diminished mRNA levels in common signature of hormone independence (Figure 2)

Entrez	Name	Title	Entrez	Name	Title
367	AR	androgen receptor	7494	XBP1	X-box binding protein 1
388	RHOB	ras homolog gene family, member B	7644	ZNF91	zinc finger protein 91
414	ARSD	arylsulfatase D	8349	HIST2H2BE	Histone 2, H2be
780	DDR1	discoidin domain receptor family, member 1	10140	TOB1	transducer of ERBB2, 1
1153	CIRBP	cold inducible RNA binding protein	10229	COQ7	coenzyme Q7 homolog, ubiquinone
1154	CISH	cytokine inducible SH2-containing protein	11201	POLI	polymerase (DNA directed) iota
1363	CPE	carboxypeptidase E	23199	KIAA0182	KIAA0182 protein
2065	ERBB3	v-erb-b2 erythroblastic leukemia viral oncogene homolog 3 (avian)	23247	KIAA0556	KIAA0556 protein
2746	GLUD1	glutamate dehydrogenase 1	25800	SLC39A6	solute carrier family 39, member 6
2804	GOLGB1	golgi autoantigen, golgin subfamily b, 1	27075	TM4SF13	tetraspanin 13
3169	FOXA1	forkhead box A1	27134	TJP3	tight junction protein 3
3382	ICA1	islet cell autoantigen 1, 69 kDa	51361	HOOK1	hook homolog 1 (Drosophila)
5241	PGR	progesterone receptor	51478	HSD17B7	hydroxysteroid (17-beta) dehydrogenase 7
5349	FXYD3	FXYD domain containing ion transport regulator 3	55930	MYO5C	Myosin VC
6509	SLC1A4	solute carrier family 1 (glutamate/neutral amino acid transporter), member 4	56654	NPDC1	neural proliferation, differentiation and control, 1

Enrichment of the prostate AI/AS gene signature within the breast cell line ER-/ER+ signature was also demonstrated using an alternative analytical technique (known as "Q1–Q2" in Tian et al., ref **15**), in which all the genes represented in the Bild breast cell line dataset were ranked by over-expression in ER- over ER+ cell lines, and the relative positions of the prostate AI and AS gene sets were each evaluated within the ranked list from the breast dataset. Over any randomly selected set of genes from the prostate dataset, and over any random assignment of the profile labels in the breast dataset, the AI genes were enriched within the ER- genes and the AS genes, within the ER+ genes (*p *= 0.0001 and *p *= 0.01, respectively). The 81 genes concurrent between AI/AS prostate cancer and the ER-/ER+ prostate signatures was termed a common signature of hormone independence. While these 81 genes represented significant similarities between hormone independent breast and prostate cancers, there were many more genes not shared between the two (Figures 1 and 2), as would expected when comparing these two rather different systems.

### Identification of a subset of clinically localized prostate tumors having the gene signature of hormone independence and showing repression of androgen signaling

Primary IBC presents as either hormone dependent (ER+) or independent (ER-). Based on the observed overlap between an *in vitro *gene signature of androgen independence in prostate cancer and the signature of estrogen independence in clinical breast tumors (Figures 1 and 2), it seemed plausible that a subset of androgen independent primary prostate tumors could be defined using the breast tumor expression profile data. Three independent mRNA profile datasets of clinically localized prostate cancer (PCA) were considered: from Glinsky *et al*. [16] of 79 tumors, from Yu *et al*. [17] of 60 tumors, and from Lapointe et al. [18] of 62 tumors. The PCA profiles in each of the three datasets were ordered based on the extent of similarity in expression patterns (by Pearson's correlation coefficient) with that of the 417 genes in the core breast ER-status signature (Figure 2).

**Figure 3:**
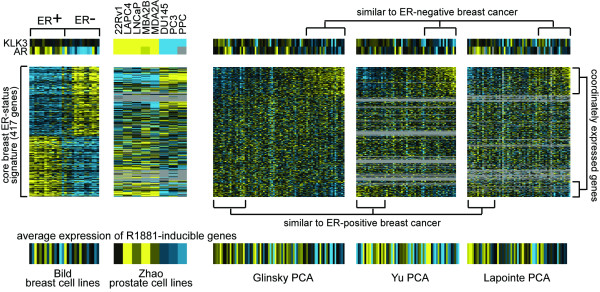
Gene expression patterns of ER- breast cancer are observed in a subset of clinically localized prostate cancer (PCA). Heat map representation for 417 unique genes differentially expressed between ER+ and ER- clinical breast tumors and cell lines (from Figure 2). The patterns corresponding to these genes are shown in three independent RNA profile datasets of PCA from Glinsky *et al*. [14], Yu *et al*. [15], and Lapointe *et al*. [16]. The PCA profiles are ordered from those that share more similarity with the ER+ breast cancer pattern to those that share more similarity with the ER- breast cancer pattern. The order of the genes is the same for each dataset represented. For each dataset, the average expression of a set of 559 genes induced by synthetic androgen R1881 *in vitro *in a dataset from Chen *et al*. [18] is also represented. The order of the breast and prostate cell lines profiles is the same as that for Figure 4. Genes and associated expression values are available in Additional File 2.

For each PCA profile dataset, a sizable fraction of the tumors were significantly correlated (*p*<0.01) with the ER-status signature (Glinsky: 52%, Yu: 52%, Lapointe: 32%), some of the PCA having patterns similar to ER- IBC, others to ER+ IBC. When selecting a random set of 417 genes from the Glinsky dataset to represent the ER-status signature, none of Glinsky tumors shared significant similarities to the random pattern as expected. These observations indicated that the set of genes associated with hormone independence in breast tumors are coordinately expressed in PCA, which was further evident when viewing the associated expression patterns as heat maps (Figure 3, associated gene expression values provided in Additional File 2). For the genes high in ER- IBC, a sizable fraction were also high in a subset of the PCA tumors; in these same tumors, a sizable fraction of the ER+ genes were down. Where the breast cancer cell lines and the PCA shared common expression patterns, the associated genes also showed concordant expression in the prostate cell line data. When comparing the subset of clinical PCA having significant similarities (*p*<0.01) to ER- breast tumors and cell lines with the subset of PCA similar to ER+ breast, the androgen receptor (AR) was significantly decreased (*p*<0.01) in PCA similar to ER- breast for the Glinsky and Lapointe datasets but not the Yu datasets. *KLK3*, which encodes prostate-specific antigen (PSA), was decreased (*p*<0.01) in PCA similar to ER- breast for the Yu and Glinksy but not the Lapointe datasets.

Transcriptional targets of the androgen signaling pathway have been defined previously using gene expression profiling of cell cultures [14,19]. From the profile dataset from Chen *et al*. [14], a set of 559 unique named genes showing induction (*p*<0.001) by synthetic androgen R1881 were obtained. Relatively few genes in the common signature of hormone independence (Figure 2) were androgen-inducible, six of them in the set of 30 AS/ER+ genes and three in the set of 51 AI/ER- genes. Of the 223 ER- genes and 194 ER+ genes in the core ER-status signature, 22 and 13, respectively, were R1881-inducible. When comparing the subset of clinical PCA having significant similarities (*p*<0.01) to ER- breast with the subset of PCA similar to ER+ breast, the PCA similar to ER- showed lower average expression of the R1881-inducible genes (Glinsky *p*<0.0002, Yu *p*<0.0007, Lapointe *p*<0.09, *t*-test, see also Figure 3). Across all of the PCA profiles, the *t*-statistic of the similarity with the ER- core signature pattern was inversely correlated (*p*<0.05, Pearson's) with the average expression of R1881-inducible genes in each of the three datasets. These patterns indicated that the androgen signaling pathway tends to be deactivated or suppressed in PCA exhibiting the gene signature of hormone independence.

### ER- breast and AI prostate cell lines are significantly enriched for transcriptional targets of the EGFR pathway

For clues as to what molecular pathways may be represented in the common gene signature of hormone independence (Figure 2), transcriptional targets of various pathways from public datasets were examined. Pathways considered included: Myc, c-Src, beta-catenin, E2F3, and H-Ras, from the expression profile dataset from Bild *et al*. [11]; Akt, from the dataset by Majumder *et al*. [20]; cyclin D1, from the dataset by Lamb *et al*. [21]; and Her2, EGFR, MEK, and Raf, from the dataset by Creighton *et al*. [22]. In the previous Creighton study, ER+ MCF-7 breast cancer cells were made to stably over-express EGFR or constitutively activate erbB-2, Raf, or MEK; which resulted in these cells exhibiting estrogen-independent growth and the down-regulation of ER expression. Of all the pathway gene signatures considered in this present study, the EGFR, MEK, and Raf signatures shared significant similarities with the common hormone independence signature. Of the 734 genes up-regulated (*p*<0.01) by EGFR, the 1238 genes up-regulated by MEK, and the 618 up-regulated by Raf: 16, 15, and 12, respectively, were shared with the 51 AI prostate/ER- breast genes of Table 1 (one-sided Fisher's exact *p*<4E-09, *p*<2E-05, and *p*<2E-06, respectively). Conversely, of the 940 genes down-regulated by EGFR, the 1182 genes down-regulated by MEK, and the 988 down-regulated by Raf: 11, 8, and 15, respectively, were shared with the 30 AS prostate/ER+ breast genes of Table 2 (Fisher's exact *p*<2E-06, *p *= 0.003, and *p*<3E-10, respectively).

**Figure 4:**
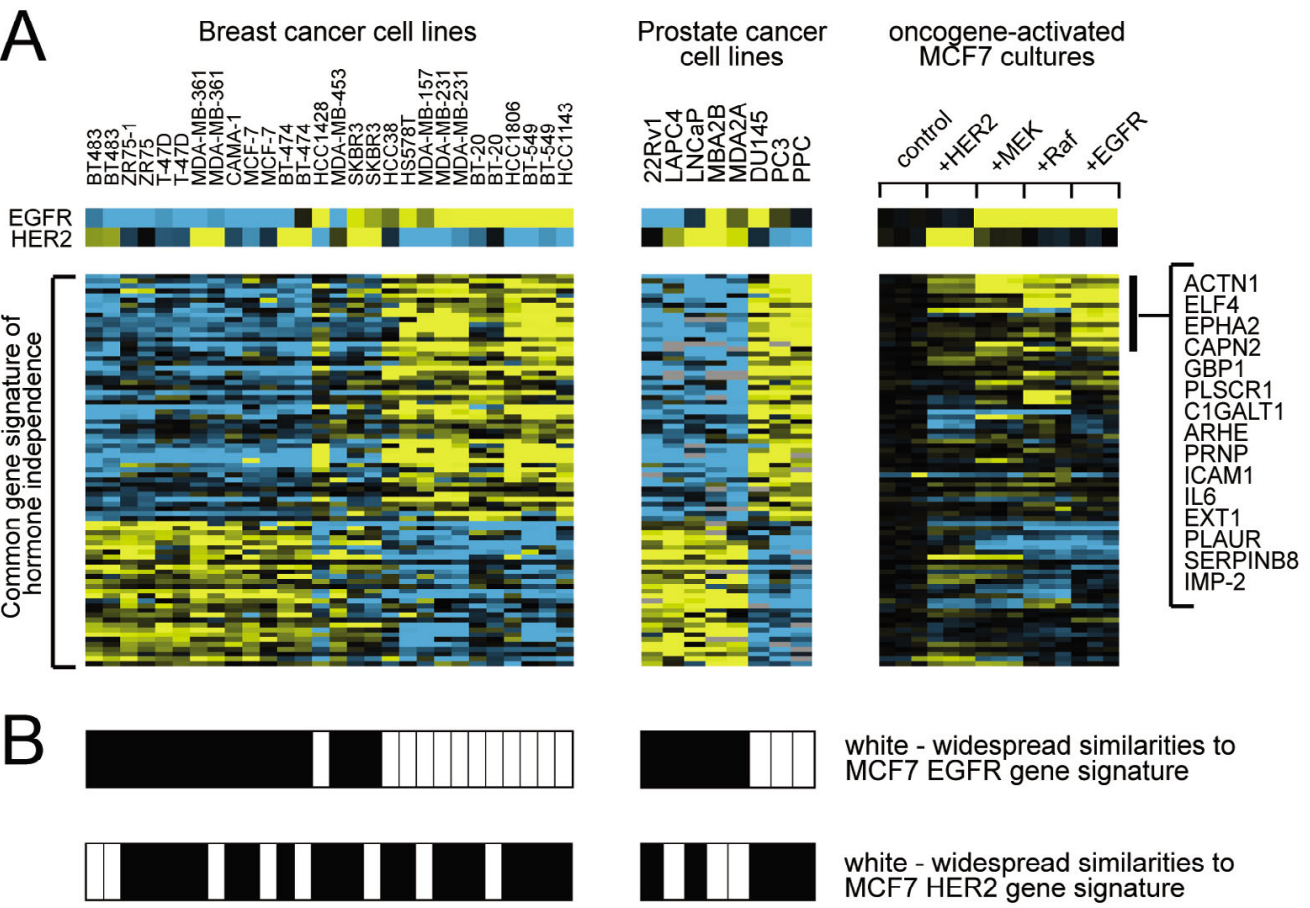
Genes associated with hormone independence in both breast and prostate cancer are enriched for transcriptional targets of the EGFR signaling pathway. **(A) **Heat map representation for 81 unique genes in the common signature of hormone independence (from Figures 1 and 2). The patterns corresponding to these genes are shown in the breast and prostate cell line profile datasets and in a dataset from Creighton *et al*. [21] of MCF-7 cell lines with activated oncogenes *HER2*, *MEK*, *Raf*, or *EGFR*. The order of the genes is the same for the datasets. The set of genes both associated with ER- and AS cell lines and activated by EGFR (*p*<0.01) are highlighted. **(B) **Classification of the breast and prostate cell lines as "EGFR-like" or "ERBB2-like," using the entire sets of genes from the Creighton dataset that were differentially expressed (*p*<0.01, irrespective of the Bild and Zhao datasets) in the *EGFR *or *HER2 *MCF-7 cell lines, respectively, as compared to controls. Genes and associated expression values are available in Additional File 3.

The expression patterns of the hormone independence signature were viewed as a heat map in the context of the patterns of the MCF7 cell lines with activated HER2, MEK, Raf, or EGFR (Figure 4A, associated gene expression values provided in Additional File 3). Most of the ER- breast tumors and cell lines over-expressed EGFR mRNA, and those that did not tended to over-expressed HER2 (Figure 2, Figure 4A). The AI prostate cell lines, however, did show over-expression of HER2 at the mRNA level. In addition, using the entire set of 1674 unique genes differentially expressed in the MCF7-EGFR cell line relative to control (*p*<0.01, 734 up-regulated), the Bild breast and Zhao prostate cell lines were stratified based on similarity (*p*<0.01, Pearson's correlation) to the EGFR gene signature pattern (Figure 4B). Most all of the ER- breast cell lines (with the exception of MDA-MD-453 and HER2+ SKBR3), all of AI prostate cell lines, and none of the ER+ or AS cell lines (with the exception of HCC1428) shared extensive similarities with the EGFR transcriptional signature. A similar analysis was carried out using the HER2 gene signature, but no stratification on the basis of hormone insensitivity was observed. The hormone independence signature showed no enrichment for HER2 transcriptional targets (Figure 4A).

## Discussion

One of the "holy grails" of both breast and prostate cancer research is to determine how these cancers acquire hormone independence and how best to treat them when they do. A number of molecular mechanisms have been postulated to explain resistance to hormone therapy in prostate cancer. One class of resistant prostate tumors continues to rely upon androgen receptor (AR) signaling through a number of means, including: over-expression of the *AR *gene, through DNA amplification or some other mechanism [14,23]; "promiscuous" point mutation in *AR*, allowing the receptor to be activated by steroids other than androgen, including anti-androgens and estrogen [24]; and ligand-independent activation of AR, mediated by oncogenes such as *ERBB2 *or *HRAS *[25]. While tumors that fall under the above may often be referred to as "androgen independent" [4], for the purposes of this present study we must draw a distinction between the above class of hormone therapy resistant prostate cancer and a second class, which bypasses AR function completely and does not rely upon androgens for growth. This second class is what appears to be involved in the gene signature of hormone independence uncovered here as being common to both ER- breast cancer and prostate cancer that is completely AI.

The specific alternative signaling pathways that allow prostate tumors to bypass AR have been somewhat elusive, one candidate pathway possibly involving *BCL2 *[4]. This present study has implicated EGFR signaling has playing an important role in bypassing AR. While transcriptional targets of EGFR were enriched in AI prostate cancer (Figure 4), *EGFR *mRNA itself did not appear elevated in AI cell lines. Neither was *EGFR *mRNA consistently elevated in the clinical PCA samples that exhibited an ER-/AI molecular phenotype (Figure 3). However, EGFR protein itself is elevated in AI over AS cell lines [26]. Recent studies have found EGFR protein expression in tumor tissues to be strongly associated with hormone refractory status [27-29]. The EGFR tyrosine kinase inhibitor gefitinib causes cell cycle arrest and initiates apoptosis in primary PCA cultures and in PCA cell lines, including DU145 and PC3 as well as LNCaP [26]. In studies using the AI cell line PC3, suppression of EGF-R signaling reduced the incidence of prostate cancer metastasis in nude mice [30].

This present study indicates that a subset of primary PCA presents as hormone independent, prior to patient treatment with adjuvant anti-androgen therapy (Figure 3). It has been previously thought that a sub-population of androgen-resistant cells might coexist with androgen-dependent cells within the tumor, and that anti-androgen therapy would therefore kill off the dependent cells and leave the resistant cells to thrive [1]. This study lends support to an adjuvant therapy strategy of combining EGFR inhibitors with anti-androgens. One question to consider in designing clinical trials testing this treatment regimen is whether PSA recurrence would be a suitable endpoint, as AI prostate cancer may not express PSA (Figure 3). The ability to identify a hormone independent subset of primary breast cancer using ER as a biomarker has implications for selecting the course of adjuvant treatment [3]. If a subset of primary prostate tumors could be diagnosed in the clinic as being hormone independent, it could warrant more aggressive treatment with alternative therapies to anti-androgens. Individual genes in the list of 81 in the gene signature of hormone independence might be good candidates for prognostic markers in PCA, or several genes in the signature could perhaps be used together.

In other studies, gene expression profiling has been carried out on hormone refractory metastases of prostate cancer [31,32]. One issue with comparing results derived from those datasets with this present study's gene signature of hormone independence is that, as discussed above, these metastases likely represent several mechanisms of hormone therapy resistance, not simply the use of EGFR-mediated bypass of AR function, as appears to be manifested in the AI prostate cell lines analyzed here. Genes expressed in the EGFR-dependent AI subtype may not be uniformly expressed in all varieties of hormone refractory cancers. The dataset from ref [32] was analyzed here in the context of the datasets used in this study; when considering the genes high in hormone-refractory prostate metastases compared to PCA, no significant overlap of these genes was observed with the genes high in the AI prostate cell lines or the ER- breast tumors (results not shown). A number of expression profiling studies using prostate tumor xenografts acquiring resistance to hormone therapy have been carried out [14,33-36]; many of these studies appear to represent cancers that develop hypersensitivity to androgen pathway activation, rather than androgen pathway independence. The hormone independent gene signature of this present study did not show coordinate expression in a profile dataset from Chen *et al*. [14] of hormone therapy-resistant prostate tumors xenografts (results not shown); as these tumor xenografts uniformly up-regulated AR, it could be presumed that these xenografts represented a model of increased sensitivity to androgen levels.

One limitation with this present study is the small number of prostate cancer cell lines for which gene expression profile data was available (five AS and three AI). Profiling studies in breast cancer indicate that there are at least two subtypes of ER- IBC, a HER2+ subtype and a "basal" subtype [6,7,12]. Interestingly, recent studies indicate that a subset of ER- breast cancer may rely upon the androgen pathway rather than the estrogen pathway [37,38], though this does not appear to represent the subset of ER- considered here, as androgen-regulated genes were not enriched in the ER- gene signature of Figure 2 (results not shown). If more prostate cancer cell lines were profiled, it might uncover additional subtypes of AI PCA to the EGFR-dependent subtype uncovered here. The basal subtype of ER- IBC also appears to rely upon EGFR signaling (Figure 4). A number of clinical trials testing the efficacy of EGFR tyrosine kinase inhibitors (TKIs) in breast cancer treatment are underway. Initial phase II studies have suggested that the EGFR TKIs do not have a high efficacy in a heavily pre-treated population of patients with metastatic breast cancer; however, in patients with hormone therapy-resistant ER+ tumors, EGFR inhibition does appear to have a significant therapeutic effect [39]. In at least one small trial, there was evidence of only minimal efficacy in advanced, metastatic ER- breast cancer [39], though one could make the conjecture that anti-EGFR therapy might be more effective in less advanced cancers.

The 81 genes in the signature of hormone independence common to prostate and breast is a much shorter set compared to the hundreds of genes associated separately with either prostate or breast (Figures 1 and 2). One may expand the set of 81 somewhat by using slightly less stringent statistical cutoffs. At the same time, the set of 81 may provide a good starting point for further study. Interestingly, a number of the 51 genes higher in AI prostate and ER- breast were previously associated with the immune response, including *ELF4*, *GBP1*, *CXCL2*, *IL6*, *IL7R*, and *IL15. IL6 *(interleukin 6) in particular – indicated here to be a transcriptional target of the EGFR pathway (Figure 4) – has been shown to promote prostate tumor growth and to play a role in the interaction between epithelial and stromal cells in prostate cancer [40]. One next step in studying these genes would likely be validation of their expression patterns in breast and prostate tissues or cell lines, using some alternative technique from microarrays, such as westerns or quantitative RT-PCR; it is expected, however, that most of the genes in the set of 81 would validate, as their expression patterns were observed in multiple profile datasets generated on different microarray platforms, which in itself could be considered validation [41].

## Conclusion

In conclusion, the hope for this study is that it may aid in the development of therapy regimens to target the subset of breast and prostate cancers that up until the present have been the most difficult to treat.

## Methods

The gene expression profile datasets used in this study were all publicly available. From the 66 PCA profiles described in the study by Yu *et al*. [15], 60 were available for this present study (collection for this dataset was facilitated by A.M. Chinnaiyan and the Oncomine team). Recent evidence emerged that the MDA-MB-435 cell line was not breast but melanoma [42] and so the MDA-MD-435 profiles in the Bild dataset [11] were removed from the analysis. Gene expression values in each dataset were log-transformed. Gene expression values in the clinical breast tumor and cell line profile datasets were centered on the centroid mean of ER- and ER+. Values in the Zhao prostate cell line profile dataset [8] were centered on the centroid mean of AS and AI. Clinical prostate tumor datasets were transformed to standard deviations from the median. For the androgen dataset from Chen *et al*. [14], expression values within the AR+ group of samples were transformed to standard deviations from the mean; values within the vector group of samples were separately transformed. Expression values in the Creighton MCF-7 dataset [22] were centered on the mean of the MCF-7/lt-E2 control group.

As the expression profile datasets were generated on different platforms, and as many of the genes represented were measured on multiple probes in any one dataset, a method to select the optimal probe to represent each gene in an unbiased fashion was used when joining multiple datasets. For the Affymetrix array datasets, the probe with the greatest variation across samples represented the gene. For the cDNA microarray datasets (Zhao cell line and Lapointe PCA), the probe with the most unflagged values across samples, followed by the probe with the greatest variation, represented the genes. The Entrez Gene identifier was used in mapping genes across datasets. Two-sample *t*-tests determined significant differences in gene expression between groups of samples. For the Chen androgen dataset, the Pearson's correlation between gene expression and the log of the concentration of R1881 determined significance of R1881 induction. Expression values were visualized as heat maps using the Cluster [43] and Java TreeView software [44]. Prior to heat map generation, genes were manually sorted using Microsoft Excel in order to highlight gene groups of interest.

The one-sided Fisher's exact test determined significance of overlap between any two distinct sets of genes. Q1–Q2 enrichment analysis [15] was carried out essentially as described in ref [45]. For "stratifying" a set of profiles in a given dataset on the basis of a pre-defined expression pattern (e.g. stratifying the clinical PCA profiles using the ER-status gene signature in Figure 3), each gene involved in the pattern was represented as "1" or "-1" (for up or down, respectively), and the Pearson's correlation coefficient was computed between the pattern and each individual profile (with significance by two-sided *t*-test).

## Abbreviations

estrogen receptor alpha (ER), progesterone receptor (PR), invasive breast cancer (IBC), clinically localized prostate cancer (PCA), androgen independent (AI), androgen sensitive (AS)

## Authors' contributions

CC conceived of the study, collected the publicly available datasets, did the analysis, and wrote the manuscript.

## Supplementary Material

Additional File 1 Supporting Data, Figure 2. Excel worksheets with the expression data that was presented as heat maps in Figure 2, along with a list of the associated genes. Click here for file

Additional File 2 Supporting Data, Figure 3. Excel worksheets with the expression data that was presented as heat maps in Figure 3, along with a list of the associated genes. Click here for file

Additional File 3 Supporting Data, Figure 4. Excel worksheets with the expression data that was presented as heat maps in Figure 4, along with a list of the associated genes. Click here for file
